# A case report of *Anisakis pegreffii* (Nematoda, Anisakidae) identified from archival paraffin sections of a Croatian patient

**DOI:** 10.1186/s12879-016-1401-x

**Published:** 2016-02-01

**Authors:** Ivona Mladineo, Marijana Popović, Irena Drmić-Hofman, Vedran Poljak

**Affiliations:** 1 Institute of Oceanography and Fisheries, Laboratory of Aquaculture, Šetalište Ivana Meštrovića 63, 21000 Split, Croatia; 2 University Hospital Centre Split, Spinčićeva 1, 21000 Split, Croatia; 3 University of Split, School of Medicine, Spinčićeva 1, 21000 Split, Croatia; 4 Health Ecology Department, Croatian National Institute of Public Health, Rockefellerova 7, 10000 Zagreb, Croatia

**Keywords:** Archival tissue section, *Anisakis pegreffii*, Croatia, Mitochondrial cytochrome oxidase 2

## Abstract

**Background:**

This is the first report of anisakiasis in a Croatian patient, evidenced from an archival paraffin-embedded and hematoxylin-eosin stained tissue section. Anisakiasis has been only suspected in the country based on previously detected anti-*Anisakis* IgE seroprevalence in the healthy coastal population, as well as an acute case where pathohistological and serological findings suggested the diseases, but the migrating larva has not been retrieved.

**Case presentation:**

Seventy years-old female, operated in 1998 for pulmonary carcinoma, was admitted to the General hospital Šibenik, Croatia in 2003, because of gastric pain and nausea that lasted for couple of days. She was showing good general condition, full mobility and lucidity, subfebrile status. Abdominal palpation inferred acute pain in paraumbilical and ileocecal region. Exploratory right pararectal laparotomy revealed a hardened, 5 cm-long structure, located intraluminally in the sigmoid colon, not perforating colon serosa. The process has been dissected and sent for patohistological diagnosis. Results showed a 2 mm-long whitish nematode spiralised in muscular layer of colon mucosa surrounded by granulomatous inflammation.

**Conclusion:**

After genomic DNA isolation of the nematode from the histological section, and amplification at the mitochondrial cytochrome oxidase 2 locus, etiological agent has been identified as *Anisakis pegreffii*. Used methodology suggests that screening of archival suspicious sections is feasible in order to study epidemiology of this zoonotic disease poorly recognised in Croatia.

## Background

The genus *Anisakis* comprises parasites with an indirect generalist character [1] and an infective third-stage larvae (L3) that is commonly found in the viscera and musculature of many teleost species [2]. Post-mortem in fish, larvae tend to migrate from the abdominal cavity where the majority is spiralised *intra vita*, into fish flesh, where they are consequently difficult to detect at plain visual inspection [3]. It has been accepted that in humans, members of the nematode family Anisakidae cause a zoonotic disease anisakidosis, whereas anisakiasis is caused by members of the genus *Anisaki*s [4]. Within genus *Anisakis*, two sibling species, *A. pegreffii* and *A. simplex sensu stricto*, are recognised as human pathogens in Europe and Japan [5, 6]. Although the first report of anisakiasis dates back to 1960 in Netherlands [7], an increase in *Anisakis* infection rate in human population has been observed nowadays, declaring anisakiasis as one of the most significant emerging food-borne diseases [8–18].

**Fig. 1:**
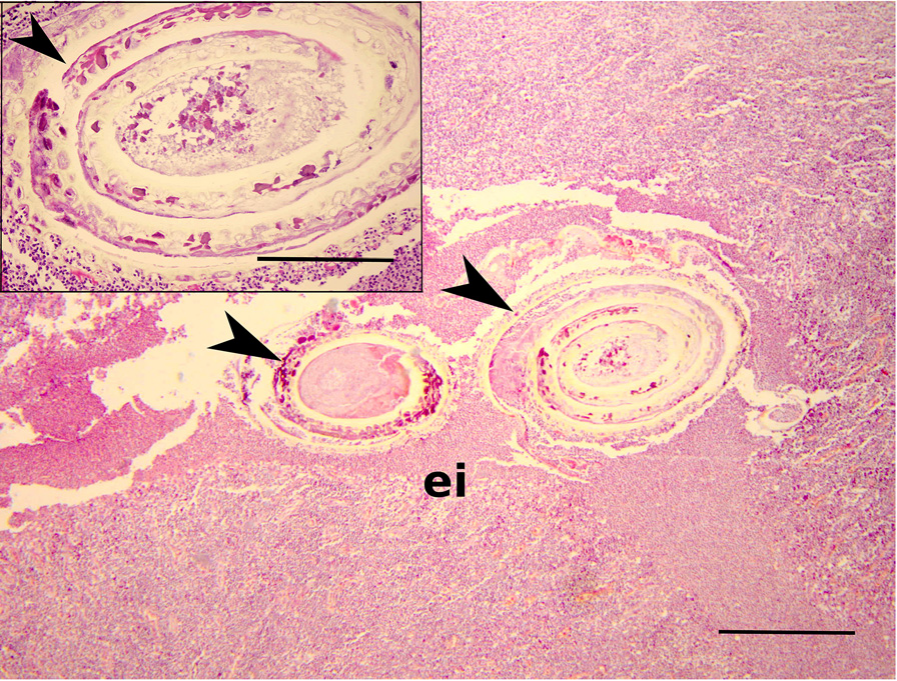
Paraffin-embedded section of the dissected patient colon showing degenerated body of the spiralised nematode (black arrowheads) and intensive eosinophilic infiltration (ei). Scale bar: 100 μm, magnification: 40x. Insert: Note the deterioration of the nematode cuticle (arrowhead). Scale bar 20 μm, magnification: 100x. H&E stained

The reasons range from purely ecological, as a more stringent conservation measures of protection of sea mammals as parasite’s final hosts, help increasing their populations and consequently that of their parasite [19]; to socio-cultural, as an ingression of “exotic” gastronomic habits throughout Europe [20]. The later includes consumption of thermally unprocessed or lightly processed traditional seafood like sushi and sashimi in Japan [21], tuna or sparid carpaccio, marinated, salted or pickled anchovy in Mediterranean [22–24], smoked or fermented herrings (maatjes) in Netherlands [25], dry cured salmon (gravlax) in Norway, raw salmon (lomi lomi) in Hawaii or ceviche in South America [26]. Finally, elevated medical consciousness to the disease and more detailed clinical examinations have enhanced the number of diagnosed cases in humans [27], although anisakiasis is still a misdiagnosed and underestimated entity in the Mediterranean.

*Anisakis* L3 larvae can enter human gastrointestinal tract at different sites of infection after ingestion of contaminated seafood, eliciting gastric, intestinal or ectopic anisakiasis [28]. Gastric anisakiasis is characterized by epigastric pain, nausea and vomits after a short period of 1–12 h postingestion of live *Anisakis* larvae [1], while in the intestinal form, abdominal pain is also the predominant symptom, but the incubation period may be delayed until 48–72 h, sometimes 5–7 days postingestion [29]. Ectopic anisakiasis relates to migration of the infective larvae through body cavities. Additionally, gastroallergic anisakiasis is another clinical form expressed in a relevant number of patients with gastric anisakiasis that have associated allergic symptoms, ranging from urticaria to anaphylactic shock [30–32]. Since allergic symptoms may predominate over gastrointestinal manifestations and most *Anisakis* infections are subclinical [22, 33], this condition can only be detected using immunological tests [34]. The quantity of cases with allergic aspects of *Anisakis* infections over Europe [23, 27, 34–36], urged recommending to carry out serological studies in populations at risk, both healthy or with food allergies in anamnesis, to understand the relevance of *Anisakis* infections in Europe [37, 38]. In south coastal part of the Adriatic Sea, Croatian population traditionally consumes home-made thermally unprocessed fish, mostly pickled, marinated, salted anchovy (*Engraulis encrasicolus*) and sardine (*Sardina pilchardus*) and the recent epidemiological study of seroprevalence of anti-*Anisakis* IgE showed that the sensitisation reached 3.5 % in the islands’ population or 2 % in the overall tested population [39]. All *Anisakis* positive subjects were high fish consumers, mostly of raw and home-made thermally unprocessed fish prepared in the traditional manner. Most of them reported professional or hobby occupational contact with fishery or fish industry. Contradictory to the anti-*Anisakis* IgE seroprevalence in Croatian costal population, no firm medical cases reporting the larva from patients have been so far evidenced in Croatia. The aim of this case report was to identify nematode larva from archival paraffin sections of a suspected case of anisakiasis using molecular tools.

## Case presentation

Seventy years-old female, operated in 1998 for pulmonary carcinoma, was admitted to the General hospital Šibenik, Croatia in 2003, because of gastric pain and nausea that lasted for couple of days. She was showing good general condition, full mobility and lucidity, subfebrile status. Abdominal palpation inferred acute pain in paraumbilical and ileocecal region. Exploratory right pararectal laparotomy revealed a hardened, 5 cm-long structure, located intraluminally in the sigmoid colon, not perforating colon serosa. In general, colon showed to be thicker on palpation, without additional processes visible by inspection. The process has been dissected and sent for patohistological diagnosis (PHD) that evidenced a 2 mm-long whitish nematode spiralised in muscular layer of colon mucosa surrounded by granulomatous inflammation, suspected as a member of *Anisakis*. PHD was done after dehydrating, paraffin-embedding, cutting sections of 5 μm thickness and hematoxylin-eosin staining of the dissected process. Granulomatous change was not encapsulated by connective tissue nor calcified, and lacked the necrotic central area, indicating an acute process. Section through intramural process revealed two asymmetrical segments of the spiralised nematode in developed state of decomposition, surrounded by eosinophils and other leukocytic cells that perforated nematode cuticle and infiltrated its polymyarian muscle cells (Fig. 1). Decomposition of the inner nematode parts did not permit to identify its hallmark structures (absence of the ventricular appendix and intestinal caecum), disabling morphological identification of the nematode.

For purpose of the molecular identification of paraffin-embedded nematode, nematode were dissected from the slides to obtain its largest area. From those sections, genomic DNA was isolated using QIAamp DNA FFPE Tissue Kit (Qiagen, Germany), for purification of genomic DNA from formalin-fixed paraffin-embedded tissues. Genomic DNA from histological sections was amplified, purified, and the mitochondrial *cox2* locus (~645 bp) was sequenced, as described previously [40]. Obtained sequence was uploaded in the Basic Local Alignment Search Tool (BLAST; http://blast.ncbi.nlm.nih.gov/Blast.cgi), and a nucleotide database using a nucleotide query was done by default parameters (database Other, Optimize for Highly similar sequences (megablast), algorithm parameters: default). Unambiguous sequence of the mitochondrial cox2 gene (stored in GenBank under accession number KU057355) revealed that the unidentified nematode from the archival paraffin-embedded and hematoxylin-eosin stained histological section belongs to *Anisakis pegreffii*. Distribution of 100 BLAST hits on the query sequence gave maximum and total score of 1085, query cover of 89 %, E value 0.0 and 100 % identity to *Anisakis pegreffii* haplotype isolated from the Adriatic Sea (KC479875).

Our results represent the first molecular confirmation of the infective *A. pegreffii* larvae from the Croatian patient that presented an acute form of intestinal anisakidosis, identified in the archival hematoxylin-eosin stained histological section of the colon. Compared to Japan and United States [4], in the Mediterranean part of Europe there is still a scarce number of reported cases of clinical anisakiasis in contrast to the significant levels of parasitation that have been observed in some of the most economically important fish species consumed in this area as thermally insufficiently processed [41–45]. In a detailed study of evaluation of the *Anisakis* risk management in the Europe, [46] estimated anisakiasis incidence of 0.038 %, averaging to 20 cases per country per year. However, epidemiological data are scattered and occasionally reported for the Mediterranean basin.

In Italy, 54 cases reported in a 5 year period (1996–2011) [46] were associated with thermally unprocessed anchovies, herring and mackerel [46–48]. Among those showing acute gastrointestinal form, usually only eosinophilic granuloma associated with larvae is observed [49], while larva itself is isolated on rare occasions [50–52]. In Spain, while the allergic aspects of *Anisakis* infections have been extensively studied resulting in hundreds of cases of allergy reported since 1995 [27, 31, 53], less information is available on acute anisakiasis. For comparison, till 1999 in Spain 9 acute cases were described [54]; in 1997 96 cases of gastroallergic anisakiasis were diagnosed in patients in Madrid [55]; while from 1999–2003, in total 65 acute cases were reported [56, 57]. Moreover, while individual accidental anisakiasis is more frequent in Europe, three massive outbreaks have been described in Spain [58–60]. In France another massive infection was reported in 6 people after consumption of raw herring. In period between 1985–1987, 21 cases were confirmed by parasitological identification and ten years later, number increased to 80 cases [61, 62]. No official records exist for other countries in the Mediterranean basin, but in general anisakiasis can be considered as an reemerging, misdiagnosed and underestimated zoonosis.

Epidemiological situation in Croatia is even less clear. A recent epidemiological study that encompassed asymptomatic rural inland, urban and island population in Mid and South Dalmatia (South-East Croatia), revealed 2 % seroprevalence [39], which is less than in Spain, Italy and Morocco [23, 62–64] and higher than in Norway [65]. Differences could be a consequence of study design (e.g. Croatian asymptomatic vs. symptomatic populations in other countries), sensitivity of the method used [34, 65, 66], as well as fish consumption per capita in each of these countries, being fairly low in Croatia (8.5 kg/year per capita; [67]). Nevertheless, the presence of circulating anti-*Anisakis* IgE in the high-risk asymptomatic coastal population, indicates that in past the population was in contact with the live larva, but the lack of anisakiasis reports further suggests that the diseases in Croatia is underestimated. Recently, a case report describing a small intestine obstruction in a 14-year-old boy was suspected as enteric anisakiasis [68]. Anamnestic data included consumption of sushi and onset of diffuse abdominal pain, nausea and vomiting 3 days later. After emergency laparotomy, inflammation and edematous changes of the terminal ileum, and enlarged mesenteric lymph nodes were observed. Histopathological analysis revealed edema and diffuse infiltration of eosinophils in muscular layer and serosa of the appendiceal wall, but no parasites at the inflammation site or in process of migration. Specific anti-*Anisakis* IgE levels were significantly increased, although authors did not report the serological test deployed or its sensitivity. Considering the absence of morphological or molecular identification of the parasite in mentioned case, as well as the possibility of false *Anisakis*-positivity of some accepted serological tests [68], much evidence is missing to relate the etiological agent to *Anisakis*.

## Conclusions

*A. pegreffii* identified from archival histopathological microscopic slides taken in 2003, morphologically could not be distinguished due to advanced processes of larval degradation. One of the main histopathological features of the local inflammatory lesions produced by infective larvae is intensive eosinophilic infiltration in the tissues surrounding the parasite, but seemingly, immune cells adherent to the larval epicuticle can not damage the nematode, instead affecting surrounding host tissue in both acute and chronic infections [69]. This suggests that anisakid larva in our case, being degraded, possibly died embedded in patient’s colon, as a plausible outcome for nematode that cannot fulfil reproductive cycle in humans. In such cases, molecular identification by PCR using *Anisakis*-specific primers is a method of choice that might be also helpful for better resolution of anisakiasis prevalence in old cases. The drawback of DNA isolated from paraffin blocks is the possibility of recovery of a degraded and fragmented DNA that would result in false-negative results [70]. In such cases, design of specific primers that amplify smaller and thus potentially intact DNA loci fragments is suggested to confirm the outcome. Previously only a single case of *A. pegreffii* infection from an Italian patient was identified from paraffin-embedded tissue [52], while this is the first case of its identification in Croatia.

## Consent

Data used in this study were from a patient deceased in 2003 from primary disease other than the one studied in this work. The research was approved by the Ethics Committee of the Croatian National Institute of Public Health (No: 001-41/1-11) based on the given consent of the next of kin.
